# Oral health in the Americas: progress, gaps, and the path to universal coverage

**DOI:** 10.1016/j.lana.2026.101458

**Published:** 2026-03-13

**Authors:** The Lancet Regional Health – Americas

As we approach World Oral Health Day on March 20, 2026, it is important to consider whether the Americas are moving towards the goals of the WHO Global Oral Health Action Plan 2023–2030. These goals include reducing oral diseases and inequalities, and integrating oral health into universal health coverage, or investigating whether the region is moving further away from these objectives.

Historically, the Americas have faced significant challenges to oral health. Estimates from the 2021 Global Burden of Disease Study suggest that populations in the Americas are among the most affected by oral conditions. These include untreated caries, severe periodontitis, edentulism, lip and oral cavity cancer, and other oral disorders. There have been substantial increases in these conditions from 1990 to 2021. During this period, untreated caries in permanent teeth increased by 52.4%, and severe periodontitis by 93.3% in the Americas. These conditions have had a substantial impact on both quality of life and disability-adjusted life years. In 2021, untreated caries in permanent teeth accounted for 296,000 disability-adjusted life years, and severe periodontitis accounted for 929,000.

If essential oral health care remains excluded from benefit packages, universal health coverage is reduced to medical care coverage rather than true health coverage. A 2024 scoping review found that oral health is poorly integrated into universal health coverage, with limited benefit packages that target only specific groups. The poor coverage of oral health highlights key structural weaknesses in health systems. It leads to some of the highest out-of-pocket spending and is rarely integrated into primary care. In many Latin American and Caribbean countries, high dental costs limit access for a significant part of the population, and access gaps are greater for rural and marginalised communities. Similar patterns are seen in the USA. Recent research found that nearly 1.7 million people lack access to dental clinics within a 30-min drive, and 24.7 million people live in dental care shortage areas, sometimes called dental clinic deserts. Rural and socioeconomically deprived communities are most affected.

Most major oral diseases, such as dental caries and periodontal disease, are non-communicable diseases. They share similar social and commercial determinants as other non-communicable diseases; including sugar, tobacco, and alcohol consumption; and socioeconomic factors. Despite associations between oral health and conditions such as diabetes, cardiovascular disease, and adverse pregnancy outcomes, oral health remains disconnected from prevention and care for non-communicable diseases. Education is an important aspect of oral disease prevention across the Americas. A study in Brazilian adolescents showed that low oral health literacy and unfavourable school environments were significantly associated with higher rates of untreated dental caries among 12-year-olds. A meta-analysis of randomised trials across seven countries suggests that school-based supervised toothbrushing interventions effectively reduce dental caries. School-based interventions, community programmes, and sustained health literacy efforts reduce caries and improve long-term outcomes. However, implementation is inconsistent and underfunded.

Brazil's Family Health Strategy illustrates the need for expanded access and sustained long-term investment. Between 2001 and 2021, the number of oral health teams in Brazil's Family Health Strategy rose from 1.9 to 29 per 100,000 inhabitants due to its implementation. The fastest growth followed the 2004 National Oral Health Policy, with an increase of 1.8 teams per 100,000 inhabitants per year. Municipalities with smaller populations, lower GDP per capita, and those in the Brazilian northeast saw the largest rates of increase. Despite this expansion, population-level indicators (such as caries prevalence and tooth loss) are still high.

To meet the WHO Global Oral Health Action Plan, countries must embed oral health across primary care and universal health coverage. This includes adding essential services to benefit packages, strengthening prevention and education, and aligning oral health with control of non-communicable diseases and regulation of commercial determinants such as sugar, alcohol, and tobacco. A significant proportion of oral diseases are preventable, socially patterned, and integral to overall health. The Americas have the evidence and infrastructure to act. Progress now requires health policies that treat oral health as a core component of health equity and universal access, with sustained and long-lasting policies.

